# Vitamin B12 and Homocysteine in Pregnant Women With Gestational Diabetes Mellitus and Their Association With Obesity

**DOI:** 10.7759/cureus.102276

**Published:** 2026-01-25

**Authors:** Manisha Sarkar, Sharbari Basu, Haritha Sagili, Sreekumaran Nair, Naveenan Ramasamy, Vetrivel Thanigaimalai

**Affiliations:** 1 Biochemistry, Jawaharlal Institute of Postgraduate Medical Education and Research, Puducherry, IND; 2 Obstetrics and Gynaecology, Jawaharlal Institute of Postgraduate Medical Education and Research, Puducherry, IND; 3 Biostatistics, Jawaharlal Institute of Postgraduate Medical Education and Research, Puducherry, IND

**Keywords:** gdm, gestational diabetes mellitus, homocysteine, insulin resistance, obesity, vitamin b12

## Abstract

Background: The role of vitamin B12 in maternal obesity, insulin resistance, and gestational diabetes mellitus (GDM) is debatable. This study was undertaken to determine and compare the levels of serum vitamin B12 and homocysteine (Hcy) in pregnant women with and without GDM and to correlate them with maternal obesity.

Methods: A total of 45 pregnant women with GDM and 45 pregnant women without GDM between 24 and 28 weeks of gestation were included in the study. Their body mass index (BMI) was calculated. Vitamin B12, Hcy, lipid profile, and blood glucose levels were measured.

Results: Serum vitamin B12 levels were low in both GDM and non-GDM groups. Only four women from the non-GDM group had normal vitamin B12 levels. Women with GDM had a significantly better socioeconomic status, probably accounting for relatively higher vitamin B12 levels, i.e., 146.2 pg/ml compared to 112 pg/ml in the non-GDM women. Women with GDM had significantly higher BMI (p = 0.001), higher triacylglycerol (TAG), low-density lipoprotein (LDL), and very low-density lipoprotein (VLDL), and lower high-density lipoprotein (HDL) as compared to non-GDM women. No significant statistical difference in Hcy was found between the two groups.

Conclusion: Vitamin B12 levels were low in both groups of women, but were higher in the GDM group, probably because the higher socioeconomic status among women with GDM played a role. Vitamin B12 deficiency was linked with higher BMI, and women with GDM had higher levels of TAG, VLDL, and blood pressure, highlighting a state of insulin resistance.

## Introduction

Various studies have shown that deficiency or insufficiency of vitamin B12 has an association with obesity, insulin resistance, and gestational diabetes mellitus (GDM) [1-5]. It is found that deficiency of vitamin B12 in adipocytes brings about changes in tissue-specific microRNAs and circulating microRNAs, which are epigenetic modulators. Hence, this adversely affects metabolic phenotype and leads to lipid accumulation [3].

Deficiency of vitamin B12 leads to alteration in levels of homocysteine (Hcy) and metabolites of one carbon cycle. Vitamin B12 has been shown to be negatively associated with Hcy [2].

GDM is found to complicate pregnancies frequently [6]. It has been proposed that the pathogenesis of glucose intolerance may involve an important role of vitamin B12, as it has the ability to regulate the synthesis of Hcy [6]. Women with GDM have been shown to have higher homocysteinemia than normal pregnant women [7]. A review article by Maher et al. reported studies that have not confirmed a link between GDM and elevated Hcy [8]. Similarly, a study from Europe did not show differences in vitamin B12 among women with GDM and those without [6].

Hence, the current study was undertaken to check the levels of vitamin B12 and Hcy in pregnant women with and without GDM and find an association with obesity, if any.

This article was previously presented as a poster abstract at the 32nd Annual Conference of Medical Biochemists of India, held at Coimbatore, India, on 12th December 2025.

## Materials and methods

The study was designed as a cross-sectional pilot study involving pregnant women. Pregnant women with and without GDM in 24-28 weeks of gestation, not below 18 years of age, were included in the study. Primigravida were diagnosed with GDM according to the International Association of Diabetes and Pregnancy Study Groups (IADPSG) criteria 2010, where a two-hour 75 gm oral glucose tolerance test (OGTT) was performed. The following cut-off values were used for diagnosing GDM: fasting blood sugar level ≥ 92 mg/dl, one-hour glucose ≥ 180 mg/dl, and two-hour glucose ≥ 153 mg/dl [9].

Obesity was determined based on the BMI calculated by the following formula: weight in kg/height in m^2^. All participating women were classified as underweight (BMI less than 18.5 kg/m^2^), normal weight (BMI between 18.5 and 24.9 kg/m^2^), overweight (BMI between 25 and 29.9 kg/m^2^), and obese (BMI over 30 kg/m^2^) based on the WHO's adjustment for the Asian population [10]. Women taking vitamin B12 supplements or steroids, and those with type 1 and type 2 diabetes, thyroid disorders, and depression were excluded from the study. Lipid profile and two-hour OGTT values were recorded from case sheets.

The sample size was calculated using the formula for comparison of two independent means. The sample size was estimated with a minimum clinically significant difference of 41 units and a pooled standard deviation of 109, with 5% level of significance and 80% power, and was calculated to be 112 in each group. It was difficult to include 112 cases and 112 controls during the study period with the ongoing pandemic, so it was decided to conduct a pilot study with 45 cases and 45 controls.

The current study was conducted in the departments of biochemistry and obstetrics & gynecology at a tertiary care center in southern India. The study was conducted after obtaining approval from the Departmental Postgraduate Research Monitoring Committee and the Institutional Ethics Committee. Written consent forms were collected from all participants at the commencement of the study.

After recruitment of participants by the clinician, 5 ml of blood was drawn under aseptic conditions. The serum was separated by centrifuging at 4000 rpm for five minutes, and samples were processed after passing a quality check. A part of it was stored at -80°C for analysis of Hcy. With the other part, serum vitamin B12 was estimated by chemiluminescence immunoassay using the UniCel DxI 600 Access Immunoassay System manufactured by Beckman Coulter, California, USA. Serum vitamin B12 levels normally range between 200 pg/ml and 900 pg/ml. Serum B12 level <200 pg/ml suggests deficiency, and serum B12 level between 200 and 300 pg/ml indicates depletion [11]. Serum Hcy was estimated by enzyme-linked immunosorbent assay (ELISA) using the Human Homocysteine ELISA kit manufactured by Abbkine, Wuhan, China. The normal level of Hcy is 5-15 micromole/L [12].

Statistical analysis was performed using SPSS version 19.0 (IBM Corp., Armonk, NY, USA). Continuous variables were expressed as mean ± standard deviation or median with interquartile range, as appropriate. Student’s t-test was used for normally distributed variables, the Mann-Whitney U test for non-normally distributed variables, and Spearman correlation was used for non-normally distributed variables. A p-value <0.05 was considered statistically significant.

## Results

Women with GDM were older, had a statistically significantly higher BMI, and their systolic blood pressure (SBP) and diastolic blood pressure (DBP) were significantly higher than those of women without GDM (Table 1).

**Table 1:** Anthropometric data of GDM and non-GDM women. Values are expressed as mean ± SD or median (IQR). Student’s t-test or Mann–Whitney U test is used. * P < 0.05 is considered statistically significant. GDM: gestational diabetes mellitus; SBP: systolic blood pressure; DBP: diastolic blood pressure.

Parameter	Cases (GDM) (n = 45), Median with IQR/mean ± SD	Controls (non-GDM) (n = 45), Median with IQR/mean ± SD	P-value
Age (years)	26 (25-29)	25 (21-26)	0.002*
Weight (kg)	69.25 ± 13.92	60.04 ± 10.76	0.001*
BMI (kg/m^2^)	28.00 ± 5.38	24.31 ± 3.93	0.001*
SBP (mm Hg)	110 (105-120.50)	102 (95.50-109.00)	0.001*
DBP (mm Hg)	72 (66.50-88.00)	64 (60-73.50)	0.001*
Income (Rs)	18000 (9500-31500)	10000 (8000-14500)	0.002*

Women in the GDM group had a statistically significantly better socio-economic status, represented by the monthly income of the head of family, compared to women without GDM. The GDM group had a median monthly income that was significantly higher than that of the group without GDM (p = 0.002).

Fasting blood glucose and one-hour and two-hour glucose values differed significantly between the two groups. Triacylglycerol (TAG) and very low-density lipoprotein (VLDL) were higher in the GDM group, while high-density lipoprotein (HDL) was low compared to the control group, though not statistically significant (Table 2).

**Table 2:** Biochemical parameters of women in GDM and non-GDM groups. Values are expressed as mean ± SD or median (IQR). Student’s t-test or Mann–Whitney U test is used. * P < 0.05 is considered statistically significant. GDM: gestational diabetes mellitus; Hcy: homocysteine.

Parameter	Cases (GDM) (n = 45), Median with IQR/Mean ± SD	Controls (non-GDM) (n = 45), Median with IQR/Mean ± SD	P-value
Fasting blood glucose (mg/dl)	99 (96.50-105)	78 (73-85)	0.001*
1-hour glucose (mg/dl)	186.71 ± 24.92	122.47 ± 23.25	0.001*
2-hour glucose (mg/dl)	155.24 ± 20.15	104.51 ± 18.73	0.001*
Total cholesterol (mg/dl)	216.04 ± 34.16	222 ± 37.05	0.382
Triglycerides (mg/dl)	225.44 ± 68.83	207 ± 67.42	0.211
Low-density lipoprotein (mg/dl)	138.27 ± 27.69	137.98 ± 27.55	0.961
High-density lipoprotein (mg/dl)	57 (50-65)	60 (54-65)	0.213
Very low-density lipoprotein (mg/dl)	42 (35-53)	37 (31-49)	0.074
Vitamin B12 (pg/ml)	146.20 (104.30-182.95)	112 (61.85-154.6)	0.045*
Hcy (micromole/L)	9.40 (7.95-11.12)	10.1 (8.09-15.16)	0.207

The median value of vitamin B12 was significantly higher in the GDM group compared to the other group (p-value = 0.045) (Table 2). Hcy levels were within normal limits in both groups, though it was higher in the group without GDM than in the group with GDM in concordance with the respective B12 values. No significant difference was noted in Hcy levels between the two groups.

Among the 90 women, vitamin B12 levels were low in 86 (95%), which included all 45 women of the GDM group. Of these 86 women, 76 (84.4%) had a deficiency (<200 pg/mL) of vitamin B12, and the remaining 10 (11.1 %) had insufficient vitamin B12 levels of 200-300 pg/mL. Only four women among those without GDM had sufficient vitamin B12 levels (Table 3).

**Table 3:** Vitamin B12 status among participants.

Vitamin B12 status	Number (%)
Deficient (<200 pg/mL)	76 (84.4%)
Insufficient (200-300 pg/mL)	10 (11.1%)
Sufficient (>300 pg/mL)	4 (4.4%)

## Discussion

A total of 45 pregnant women with GDM and 45 pregnant women without GDM, in their second trimester, were selected for the study. The median age of women with GDM was 26 years, as against 25 years for women without GDM. Studies by Sukumar et al. and Knight et al. also reported that women with GDM were older than those without GDM [1,13].

Vitamin B12 insufficiency and deficiency were found to be very common among both groups of pregnant women. A total of 84.4% of women had a deficiency of vitamin B12 (<200 pg/mL). This was much higher than that reported in other studies, i.e., 26% with a sample size of 344 in the United Kingdom [1] and 43% with a sample size of 785 in Mysore, India [5], conducted earlier in 2016 and 2009, respectively. Similarly, the study conducted by Sayar et al. with a sample size of 250 showed that 90.4% of Turkish pregnant women had vitamin B12 deficiency [14].

The median value of vitamin B12 in the GDM group was 146.20 pg/mL, while that in controls was lower at 112 pg/mL (p-value = 0.045). Similar to our study, studies by Radzicka et al. [6], Tarim et al. [7], and Seghieri et al. [15] showed that serum vitamin B12 levels in the GDM group were higher than those in the non-GDM group, but they did not differ significantly. On the contrary, a study by Sukumar et al. and some studies in the meta-analysis by Kouroglou et al. have reported lower levels of vitamin B12 in the GDM group than in controls [1,3]. In the current study, it was observed that women with GDM belonged to a better socio-economic class, and probably due to better nutrition, had increased vitamin B12 levels compared to the control group. In the Pune Maternal Nutrition Study (PMNS), it has been suggested that the imbalance of vitamins may be contributed to by religious and socioeconomic factors and medical practices [16]. Halicioglu et al. suggest that among pregnant women with low incomes, vitamin B12 deficiency is more common and is influenced by socioeconomic and cultural variables. Vitamin B12 status seems to be significantly impacted by the dietary consumption of animal products. Programs for food supplementation or fortification may be taken into consideration to avoid vitamin B12 deficiency [17].

In the current study, a negative correlation was observed between fasting sugar and vitamin B12 levels in both GDM and non-GDM women, though not statistically significant (Table 2). Hence, controversy continues to exist regarding the association between vitamin B12 and the risk of GDM. A meta-analysis by He et al. reports that the relation between risk of GDM and deficiency of vitamin B12 is controversial [4]. GDM risk increases when there is low vitamin B12 and high folate, and this may be found in women who take folate in excess of the recommended dosage [8]. In our study, although folate levels were not measured, most participants were on supplementation with folate. The actual mechanism linking the ratio of folate and vitamin B12 with the risk of GDM remains uncertain [8].

In the current study, the group with GDM women had a significantly higher bodyweight and BMI (Table 1) as compared to those without GDM (p = 0.001), and a negative correlation was found between BMI and vitamin B12. This is in concordance with a study from India where women with a deficiency of vitamin B12 had higher BMI and higher levels of folate [5]. The authors have opined that adiposity may be promoted by a deficiency of vitamin B12 and that adiposity may reduce vitamin B12 levels during pregnancy due to hemodilution, urinary loss from increased glomerular filtration rate, or transfer of nutrients to the growing fetus [5]. In another study by Knight et al., lower vitamin B12 was associated with higher BMI in non-GDM women. They hypothesized that this could be because of obesity in pregnancy itself and not due to lifestyle, sociodemographic, or hemodilution effect [13].

Increased levels of Hcy are a marker of vitamin B12 and folate deficiency and also of cardiovascular risk, insulin resistance, and diabetes [5]. In the current study, the median value of Hcy in the group with GDM was 9.4 µmol/L, while that in the other group was 10.1 µmol/L, and the values between the two groups did not differ significantly and were within the normal range. Some studies have shown Hcy to be higher in GDM, while one study did not find much difference [3,6]. In a study by Radzicka et al., serum Hcy did not differ between the GDM and control groups, which is similar to our study [6]. The study conducted by Liu et al. in China showed that trimester-specific serum Hcy reference intervals for pregnant women were 4.35~10.16 μmol/l in the first trimester, 3.38~8.60 μmol/l in the second trimester, and 3.75~11.17 μmol/l in the third trimester [18]. Similarly, in studies conducted on Canadian, Spanish, and Dutch pregnant women in their second trimester, Hcy levels were 4.3 ± 1.0 μmol/l, 5.22 ± 1.29 μmol/l, and 7.33 μmol/l (4.25~12.64 μmol/l), respectively [19-21]. The low Hcy level is associated with an altered maternal amino acid metabolism driven by fetal requirement, hemodilution caused by the increased blood volume, and elevated glomerular filtration rate. The fetus may also absorb a proportion of Hcy during pregnancy [18]. Hcy levels are decreased during pregnancy due to increased estrogen levels and as a physiological response to pregnancy [7]. The Hcy levels in both groups in our study were within physiological limits. In comparison with the values of Hcy in pregnant women in the above studies [18-21], women of both groups in the current study had hyperhomocysteinemia (HHcy), which may be associated with vitamin B12 deficiency.

It is postulated that in vitamin B12 deficiency, folate is trapped and synthesis of methionine is hampered, which in turn affects protein synthesis and deposition of lean tissue. Also, methylmalonic acid cannot be converted to succinyl-CoA, and the excess methylmalonic acid may lead to fatty acid synthesis, adiposity, and insulin resistance [5,13].

In the current study, not much difference has been found in the lipid profile of both groups, though TAG, low-density lipoprotein (LDL), and VLDL levels are higher, and HDL is lower in the group of women with GDM, who have a higher BMI and weight. Defect in insulin action is known to be associated with hypertriglyceridemia, low levels of HDL, and high blood pressure [3], and these match the findings in the current study. Vitamin B12 levels have correlated inversely with serum TAG in the study by Radzicka [6], but no such correlation was found in the current study. Women with GDM had low vitamin B12 levels and also had features of insulin resistance, which is similar to the study by Krishnaveni et al. [5]. The study by Adaikalakoteswari et al. showed that B12 deficiency in adipocytes causes excess accumulation of lipids and increased expression of genes that regulate adipogenesis and lipogenesis, and also changes the expression of miRNAs involved in key metabolic pathways, which may lead to insulin resistance [22].

Hence, it was observed that vitamin B12 was low in pregnant women in both groups and significantly lower in women without GDM compared to that of women with GDM. Women with GDM had obesity and a deranged lipid profile. Hcy levels, though in the physiological range in both groups, are higher than the range seen in pregnant women in the second and third trimesters. Since the incidences of obesity and GDM are on the rise and predispose to insulin resistance and its associated complications, and studies point toward an association with vitamin B12 and folate levels in serum, further studies with larger samples should be conducted so that maternal supplementation of micronutrients like vitamin B12 and folate can be re-examined.

Limitations

The sample size was small as it was a pilot study. Folate levels and the folate-to-vitamin B12 ratio would have better highlighted the role of vitamin B12 and folate in GDM. Details of dietary patterns could not be taken into account. Vitamin B12 levels in a group of non-pregnant women could have been further studied.

## Conclusions

Vitamin B12 levels were low in both groups of women, but the GDM group showed a relatively high level of vitamin B12. They also belonged to a better socioeconomic class. Women with GDM were older, obese, and had higher levels of serum glucose as compared to the non-GDM group. Vitamin B12 deficiency is linked with higher BMI and features of insulin resistance in the group of women with GDM. Hcy did not differ much between the two groups in this study.
